# Nurses experiences regarding ideal clinic project implementation in eThekwini district

**DOI:** 10.4102/hsag.v29i0.2407

**Published:** 2024-05-31

**Authors:** Thembelihle S.P. Ngxongo, Mthokozisi Zulu

**Affiliations:** 1 Department of Nursing, Faculty of Health Sciences, Durban University of Technology, Durban, South Africa

**Keywords:** clinic, ideal clinic project, implementation, KwaZulu-Natal, nurses, primary health care

## Abstract

**Background:**

The South African National Department of Health introduced the ideal clinic realisation and maintenance (ICRM) programme in response to primary health care (PHC) services and to lay a strong foundation for the National Health Insurance implementation. The progress report 2015–2016 on the implementation of this programme indicated that achieving the 50% target in selected vital areas such as staffing, resource allocation, and utilisation was not achieved.

**Aim:**

The study aimed to explore and describe nurses’ experiences regarding the ideal clinic project (ICP) implementation.

**Setting:**

The study was conducted in 18 PHC clinics in eThekwini district, KwaZulu-Natal.

**Methods:**

An exploratory descriptive, contextual qualitative design was employed guided by Donabedian’s structure, process, and outcomes model. Data were collected using semi-structured interviews with 24 nurses between 15 September and 25 October 2020 following receipt of ethics and analysed using Tesch’s open coding approach.

**Results:**

The three themes that emerged included structural limitations, processes involved in running the clinic, and support offered to the PHC clinics. These were highlighted as challenges experienced by nurses during the implementation of an ICP.

**Conclusion:**

Nurses’ negative experiences and perceptions made it difficult for them to accomplish the ICP standards.

**Contribution:**

The findings from the study highlighted critical actions by the health care institution management which if instituted, could facilitate improved implementation of the ICP and achievement of the 50% target in selected vital areas.

## Introduction

The key outcome of the vision of the South African government is the attainment of a long and healthy life for all its citizens (Republic of South Africa, Department of Health [DoH] 2014). In view of this, the government of South Africa has embarked on a phased implementation of National Health Insurance to achieve universal health coverage to provide access to appropriate, affordable, and efficient quality health services for all (Republic of South Africa, DoH 2014). In their description of the referral system and levels of healthcare, the KwaZulu-Natal (KZN) Department of Health (n.d.) describes a primary health care (PHC) clinic as the first step in the provision of health care that offers services such as immunisation, family planning, antenatal care, treatment of common diseases, treatment and management of tuberculosis, HIV counselling, among other services. Primary health care services evolution arose from the indigenous people often being excluded from mainstream health care services. The success of the PHC services lies in the fact that they include comprehensive programmes that incorporate treatment and management, prevention and health promotion, as well as addressing the social determinants of health (Harfield et al. 2018). When the new Constitution of South Africa was adopted in 1996, every South African was guaranteed access to health care (Republic of South Africa, DoH 2015). In addition, there has been a drive globally to improve patient experience through service redesign and good information provision (Thomson, Rivas & Giovannoni 2015). Thus, the Ideal Clinic Realisation and Maintenance (ICRM) programme was formed in response to the current shortages of PHC services and to lay a strong foundation for the implementation of National Health Insurance (Hunter et al. 2017). The ICRM initiative hopes to transform PHC in line with broader national priorities as laid out in Chapter 10 of the National Development Plan 2030 and aspires to ensure that each of the 3507 PHC facilities displays the elements of an ‘ideal clinic’ (Republic of South Africa, DoH 2015). The ‘ideal clinic project’ (ICP) is an initiative that was established in July 2013 as a way of systematically improving and correcting shortages in the PHC clinics in the public sector (Republic of South Africa, DoH 2016). An ideal clinic is defined as a clinic with good infrastructure, adequate staff, adequate medicine and supplies, good administrative processes, and adequate bulk supplies (Republic of South Africa, DoH 2014). The South African DoH (2014) emphasises that an ideal clinic uses applicable clinical policies, protocols, and guidelines, and it harnesses partner and stakeholder support and also collaborates with other government departments, the private sector, and non-government organisations to address the social determinants of health.

The ICP initiative was established as a way of improving the status of PHC clinics, the quality of services, and staff establishment in PHC clinics (Republic of South Africa, DoH 2016). It was developed to address gaps in PHC clinics and establish an algorithmic approach to change all the PHC clinics to adhere to the National Health Insurance standards (Republic of South Africa, DoH 2016). The South African target was for all PHC clinics to achieve 50% of the set standards in selected vital areas such as staffing, resource allocation, and utilisation, to name a few, by 2019. According to Hunter et al. (2017), only 24% (121 out of 600) of PHC clinics in South Africa had been declared as ideal clinics, with several PHC clinics still scoring low in these vital areas by 2017. Furthermore, many patients continued to bypass the PHC clinics to attend hospitals for their initial contact visits because of overcrowded PHC clinics, long waiting times, lack of sufficient medication, inadequately trained personnel, negative staff attitudes, and poorly structured and inaccessible PHC clinics. This resulted in a waste of time and resources because hospitals needed to attend to less ill patients who might have been seen and treated at local PHC clinics instead of dedicating hospitals to patients who needed them the most (Republic of South Africa, DoH 2014). These findings were evidence that there were some problems in the implementation and maintenance of the ICP in the eThekwini district. Thus, the researchers intended to explore the experiences of nurses in the implementation of the ICP in this district.

The Donabedian model (1966) was used as a theoretical framework to guide this study. The model describes the information about the quality of care in three categories: ‘structure’, ‘process’, and ‘outcomes’ (Donabedian 2003). Similarly, the national DoH proposed several components and sub-components that need to be fulfilled to achieve an ‘ideal clinic’ status which include structures and processes in the PHC clinics which are intended for quality assurance of health care services (Republic of South Africa, DoH 2016). In this study, the researchers focused on the experiences of nurses related to the structure which included the factors that affected the context in which the nurses were required to implement the ICP, and various technical and interpersonal processes undertaken during this process. All this informed the quality of care provided to patients as the intended outcome of the ICP. Through this exploration, the researcher was able to gain a deeper understanding of the perception of nurses regarding the implementation of an ICP, the support received, and the challenges they faced during the implementation of this project. This led to the determination of the strategies that can be instituted to facilitate the successful implementation of an ICP. The model also guided the presentation and interpretation of findings and structuring the recommendations from the study in a manner that can facilitate implementation thereof.

## Research aim and objectives

The study aimed to explore and describe the experiences of nurses regarding the implementation of an ICP in the eThekwini Health district in KZN.

The researchers hoped to identify through this study, the successes made, challenges experienced, and the factors that influenced the implementation of the ICP. These findings could be useful in policy formulation and amendment and could play a pivotal role in the creation of new protocols aimed at improving the quality of health care.

## Research methods and design

An exploratory descriptive, contextual qualitative design was employed. The qualitative design assisted the researcher in exploring the experiences of nurses regarding the implementation of the ICP and to provide a thick description of the experiences, perceptions, and challenges faced by nurses, support given to nurses, and strategies which can be used to influence successful implementation of an ideal clinic. The researcher adopted a naturalist paradigm, sometimes referred to as constructivist paradigm. The naturalist paradigm is mostly allied to qualitative research and assumes that reality is not a fixed entity but rather a construction of the individual participating in the research (Botma et al. 2010; Creswell 2014). The researcher’s position was that the reality regarding implementation of an ICP can be constructed through the information provided by nurses who are involved with this implementation. The characteristics that influenced this research were mainly the principal researcher’s qualifications and experience. The researcher is a registered Nurse and midwife with over 10 years of working experience as a PHC nurse and has been working in a PHC clinic which has been part of the ICP from the inception of the project.

### Setting

The study was conducted in the eThekwini Health district in KZN. Primary health care clinics attached to one of the 18 government-owned hospitals in eThekwini district were included. The vision for this hospital is to provide optimal health care to all patients in the catchment area. Much emphasis is being put on PHC which is being rendered by the satellite clinics attached to the hospital. At the time of the study, the hospital had 18 PHC clinics and a number of other types of health care clinics such as mobile clinics, community health care centres, and midwife obstetric units attached to it which were referred to as satellite or feeder clinics. These clinics were all located in eThekwini district and their operation was managed from the said hospital. Although most PHC clinics provide a comprehensive package of services, the health care services provided differ from clinic to clinic but mostly include preventive, promotive, curative, and rehabilitative services such as child immunisation, minor ailments, maternal and child care services, and chronic care. All 18 PHC clinics were included in the study as they met the inclusion criteria. The researchers used census sampling of all PHC clinics to gain a complete picture of the implementation of the ICP in this setting. All other types of health care clinics were excluded because these had different operations to that of PHC clinics.

All PHC clinics were involved in the implementation of the ICP and from time to time, usually at six monthly intervals, were assessed for compliance to the specification of the project regarding the progress and sustainability of the ICP. This process was referred to as ICRM programme. It was one of the PHC re-engineering interventions. The concept made provision for continuous and robust quality improvement principles aligned with the National Core Standards, which are the South African Presidential priorities that provide a systematic quality improvement approach to fast-track improvements on efficiencies and quality at PHC clinics (Republic of South Africa, DoH 2014).

### Study population and sampling strategy

The population of this study comprised all the nurses who had been working in the PHC clinics for 3 months or more. All categories of nurses, namely, operational managers, professional nurses, enrolled nurses, and enrolled nursing assistants, were included. The inclusion of different categories of nurses ensured that an in-depth and wide range of information was gathered as different categories had varying scopes of practice and therefore would have experienced the implementation of the ICP differently. Non-nursing employees working in the selected PHC clinics and all nurses who have been working in the selected PHC clinics for less than 3 months were excluded. Sampling for nurses was purposive in that only nurses who met the inclusion criteria were included. Sampling was based on the availability and willingness to take part in the study. The sample for nurse participants was guided by data saturation which was reached after 18 interviews and confirmed with six additional interviews thus, making a sample of 24 participants.

### Data collection

The researcher arranged a private room in each PHC clinic to ensure that there was privacy and no disruption during the interview. The researcher who is a registered nurse and therefore well-conversed with the use of personal protective equipment ensured that the room was big enough to allow social distancing according to the coronavirus disease 2019 (COVID-19) prevention protocol, and precautions were adhered to with regards to the seating arrangement, ventilation, use of sanitiser, and the wearing of protective devices such as face masks during each interview session.

One-on-one semi-structured interviews were used to collect data from the participants. The interviews were conducted by the researcher in English over a period of 4 weeks (15 September–25 October 2020) with each interview session lasting for 30–45 min. The interviews were guided and semi-structured using an interview guide to ensure uniformity of interviews for all participants. A similar interview guide which was developed by the researcher was used for all the categories of nurses. The guide consisted of five main questions each of which was aligned to particular research objectives. Furthermore, probing questions which were not predetermined but were dependent on the need for clarity or more information required based on responses by the participants to the research question were asked. The probing questions were based on the three broad elements of the model guiding the study which were structure, process, and outcome.

The researcher alternated data-collection events between PHC clinics to ensure that all 18 PHC clinics were included. One interview was conducted per clinic per day to allow concurrent data analysis to keep track of data saturation before gathering more data and also to ensure that all 18 PHC clinics were included. All interviews took place in the PHC clinics. All interviews were voice recorded with consent from the participants. Field notes were also taken to substantiate the recorded information and to capture non-verbal cues.

A minimum of one and a maximum of two interviews were conducted in each of the 18 PHC clinics.

### Data analysis

Data analysis was done concurrently with data collection, immediately the same day or within a day or two after collection and before moving to the next PHC clinic to conduct more interviews. This facilitated the monitoring of data saturation and ensured a better comprehension of the information gathered while the interview session was still fresh in the researcher’s mind. The first step during data analysis was to listen to the voice recorded information, read the field notes and compare these two data sources several times until both were fully understood. This assisted the researcher to get a clearer understanding of the information. The audio-recorded information was transcribed verbatim into a written format and again compared and read against the field notes for clarity. The transcribed interviews were captured onto a master file through Microsoft Word and thereafter Tesch’s (1992) open coding approach was used to analyse the information, which involved the eight steps which began from reading through all the transcripts to get a general impression of the collected data up until themes and subthemes were identified and grouped together.

### Ethical considerations

Data collection commenced only after full ethics approval had been granted by the Durban University of Technology Institutional Research Ethics Committee (IREC 085/20) and gatekeeper’s permission granted by the Provincial and District Department of Health Research Committees, and the Hospital Chief Executive Officer. All participants had to sign informed consent after being fully orientated about the study.

### Measures of trustworthiness

The four criteria of credibility, transferability, dependability, and confirmability described by Lincoln and Guba as cited by Stahl and King (2020) were applied to ensure trustworthiness. Using diversity in the selection of the participants whereby census sampling of all PHC clinics was ensured and data were collected from different categories of nursing personnel were the attempts to increase the credibility of data. Although there are reservations about the transferability of findings of qualitative studies, the researcher ensured that other researchers could build on the findings of this study when performing further research by providing a deep description of the research setting, and completely and constantly recording the activities about data gathering and analysis and all other research processes. This process also ensured the confirmability and dependability of the study. Confirmability was also assured by using direct quotations taken from the transcripts to present findings from the study.

## Results

### Participants’ demographic information

Data regarding the demographic characteristics of the study participants were collected before each interview session. The findings of this data were quantified to facilitate interpretation and better understanding of it. The results gathered are presented in Table 1.

**TABLE 1:** Demographic characteristics of the study participants (*N* = 24).

Demographic variable	Number	%
**Gender**		
Female	24	100
Male	0	0
**Age group (years)**		
< 25	0	0
25–35	8	33
> 35	16	67
**Category**		
Operational managers	4	17
Professional nurses	12	50
Enrolled human immunodeficiency virus	6	25
Enrolled nursing assistants	2	8
**Experience as a nurse (years)**		
< 2	0	0
> 2	24	100
**Duration of working in the PHC clinic (years)**		
< 2	0	0
> 2	24	100

PHC, primary health care.

### Themes and sub-themes

As presented in Table 2, three themes and several subthemes emerged from the interviews.

**TABLE 2:** Themes and subthemes that emerged from the interviews.

Themes	Subthemes
1. Infrastructure limitations	1.1 Physical infrastructure 1.2 Provision of equipment 1.3 Staff establishment in PHC clinics
2. Processes involved in the running of the PHC clinic	2.1 Documents and records used in the PHC clinics 2.2 Too much paperwork
3. Support offered to PHC clinics	3.1 Support from each other and between clinics 3.2 Support from management and the District office

PHC, primary health care.

#### Theme 1: Infrastructure limitations

In addition to the physical infrastructure, other supporting elements such as equipment, patient access to health care in PHC clinics, information technology, systems and processes, sustainability initiatives, and staff form part of the PHC clinic infrastructure. All these elements of the infrastructure are needed for any existing health care system to provide service delivery timeously and to facilitate positive patient experience, effectiveness, efficiency, timeliness, safety, equity, and sustainability of health care services delivery all of which are quality domains (Luxon 2015). The participants highlighted that limitations related to three elements of the infrastructure, namely, staff establishment, provision of equipment, and physical infrastructure were the main hindrances in their endeavour to implement ICP. This was evident in the following statements by some of the participants:

‘The ideal clinic project is a frustrating initiative as there are several structural limitations which do not allow for the three streams to run accordingly.’ (PHC C, Participant 2, 33 years)

‘There are many challenges that we are facing, in my opinion, most clinics are not structurally suitable to implement the ideal clinic projects.’ (Participant 4 from PHC. [PHC R, Participant 2, 29 years])

It is evidenced in research that infrastructure is important in supporting the fundamental aim of promoting improved standards of care and wellbeing for all patients, and a good experience of the health care system by health care users. Nonetheless, the healthcare system challenges that South Africa faces are systemic and structural challenges which include widespread inefficiencies, staff shortages, variability in skill sets between rural and urban areas, and suboptimal care levels and patient management (De Villiers 2021).

**Sub-theme 1.1: Physical infrastructure:** Adequate physical infrastructure is needed for any existing health care system to provide service delivery timeously. The participants of this study indicated that the infrastructure posed a major problem in ICP implementation. The infrastructure was old with some on the brink of collapse posing dangers to both staff and patients and not meeting the ICP standard. This view was evident in the following statements by some of the participants:

‘The biggest challenge with the ideal clinic implementation is that the infrastructure is congested and sometimes patients wait in the queue outside the facility for a long time awaiting service delivery.’ (PHC G, Participant 1, 40 years)

‘There are many challenges that we are facing, for example, infrastructure is not conducive towards the implementation of the ideal clinic.’ (PHC A, Participant 1, 29 years)

‘The challenge is infrastructure, for example, the consulting rooms are too small.’ (PHC M, Participant 2, 34 years)

Researchers like Oyekale (2017) attest to the fact that many of the requirements for a good infrastructure are absent in many developing countries. This compromises the quality of healthcare services and hinders the effective administration and management of patient care. According to Oyekale (2017), poor maintenance of old infrastructure, and inability to provide sufficient funds in order to replace old structures are some of the factors that contribute to proper infrastructure.

**Sub-theme 1.2: Equipment supplies:** Medical equipment is a necessary tool for nurses to provide quality nursing care and its shortage has a negative impact on patients, the healthcare facility and the nursing profession, and a barrier for the health system to function. Nonetheless, access to functioning medical equipment is a challenge in low and middle-income countries (Moyimane, Matlala & Kekana 2017). Several issues related to equipment used daily which ranged from the unavailability, malfunctions and obsolete models of equipment which often resulted in nurses skipping some aspects of nursing care were shared by the participant. The excerpts discussed further are a few examples of how this notion was shared by the participants:

‘There is lack of needed equipment for service delivery, therefore there is stagnation in Ideal Clinic implementation.’ (PHC D, Participant 2, 42 years)

‘Since we are ideal clinic, we are facing challenges such as a shortage of equipment in the facility which gives us huge problems because patients expect service delivery in time which cause a lot of frustration.’ (PHC G, Participant 2, 25 years)

Shortages of medical equipment in health care facilities including PHC clinics often result from malfunctioning equipment, poor maintenance, and unavailability of equipment caused by budgetary constraints and form a barrier to the ability of the health system to deliver quality health services (Moyimane et al. 2017).

**Sub-theme 1.3: Staffing of the facility:** Nurses have long been central to health care, especially in rural areas where physicians are reluctant to practise and also in PHC clinics where healthcare services are nurse-driven (Mayosi & Benatar 2014). Some of the participants stated that their clinics were not adequately staffed, thereby predisposing them to work overload and burn out. All this in turn exposes them to skip a lot of fundamental elements that comprise ICP implementation. This was evident in the following statements by some of the participants:

‘There has been a few glitches when it comes to availability of resources and staffing in order for the programme to run.’ (PHC H, Participant 2, 31 years)

‘We are short staffed, other nurses have to go as far as to pulling of files which is a duty that is intended for admin clerks.’ (PHC C, Participant 1, 28 years)

Abrahams, Thani and Kahn (2022) attest to the fact that challenges such as understaffing are common in South Africa with PHC clinics often being the most poorly resourced. Shortage in the staff in health facilities does not only refer to the number of staff, but a shortage of competent and scarce-skilled health professionals as this leads to difficulty in maintaining the quality of healthcare standards (Abrahams et al. 2022).

#### Theme 2: Processes involved in the running of the primary health care clinic

Some processes under the ICP are required to be adhered to for the ideal clinic to become a reality. These processes are described in the manual as part of the framework used for tracking the progress of the PHC clinics in implementing ICP (Republic of South Africa, DoH 2020). According to the study participants, the processes that were required to be adhered to for the ICP were not always achievable nor in place in the clinics for a number of reasons which included either staff members being clueless of what was expected of them, being short staffed, or the equipment required for implementation of these processes not available. Some of the statements shared by the participants in this regard are discussed further:

‘I always believe that it is important that processes are defined and made known to us as staff otherwise it becomes too difficult to do things correctly where there are no defined processes it.’ (PHC C, Participant 2, 33 years)

‘Some of the things that we are expected to do are impossible, they create processes that are complex or sometimes not aligned to the current situations in most PHC clinics.’ (PHC P, Participant 1, 44 years)

According to Donabedian (2003), the measurement of process is nearly equivalent to the measurement of quality care. Thus, where processes are not adhered to, quality is often compromised.

**Sub-theme 2.1: Documents and records used in the primary health care clinics:** A number of documents are used by nurses during healthcare services provision. These include policy documents and clinical guidelines used to guide practice, clinic registers used to record patient information, and data-collection sheets used to capture clinic statistics. The participants indicated challenges in the supply and use of these documents. There were challenges with the dissemination of new policies and clinical practice guidelines intended to guide nurses in delivering care. These were often not delivered to the PHC clinics and staff ended up downloading these at their expense or if not downloaded, they ended up using old and outdated documents which were different than what was intended by current practice. In addition, registers and tally sheets were not aligned, thus resulting in a lot of confusion and distortion of information especially when it was time to summarise statistics for reporting. The confusion caused by these discrepancies resulted in slowing down of daily operation as the staff struggled to ensure accurate completion of these documents. These concerns regarding documents were evident in the following statements by some of the participants:

‘We do not have guidelines that we are supposed to have in the facility but we end up downloading them with our phones instead.’ (PHC J, Participant 1, 27 years)

‘The challenge we have is with the tally sheet as they do not correspond with the tick registers that we use on a daily basis.’ (PHC I, Participant 1, 29 years)

When policies and guidelines are developed and/or implemented according to international standards, they have the potential to reduce unwarranted practice variation, enhance translation of research into practice, improve healthcare quality and safety, and have the potential of influencing care outcomes, provided they are effectively disseminated and implemented (Panteli et al. 2019).

**Sub-theme 2.2: Too much paperwork:** Traditionally, the majority of PHC clinics in South Africa use paper-based records, yet completing these records is time consuming. The participants reported that paperwork on patients’ files was too much to bear; there were a lot of registers and a lot of information that needed to be added in patients’ files and registers appeared as unnecessary duplication. The following are some of the statements shared by the participants in this regard:

‘There is a lot of paperwork as opposed to nursing the patient, for example there are now a lot of registers and duplication of information thus resulting in a long waiting time for the patient.’ (PHC H, Participant 2, 31 years)

‘For now, too much time is spent on paperwork; I think the reduction of paperwork can really help and can also improve waiting times. This can also help in improving patient satisfaction and service delivery.’ (PHC F, Participant 1, 36 years)

Tinggi et al. (2021) advises that the workload should be within comfortable limits for the employees who form part of the organisation. When the workload exceeds employee capabilities, they often experience pressure from the work undertaken and this has a negative impact on their performance which can result in less efficiency at work (Rizky & Fetty 2023).

#### Theme 3: Support offered to primary health care clinics

Support at work is an expectation by every employee and an important component when it comes to enhancing employee performance. The participants commented a lot on the support that they get and the support that they should be getting in the ICP but they were either not getting or it is not uniformly given to all staff. The following were some of the statements shared by the participants in this regard:

‘What has also impacted on the implementation of ICP is the nature of support that is available to staff and clinics which is not uniform such that for other clinics is non-existent.’ (PHC G, Participant 1, 40 years)

‘For me support is the key, there is no way that staff and clinics can succeed without support.’ (PHC O, Participant 1, 26 years)

‘If only there could be support things will be much easier, for now we struggle alone without much support.’ (PHC R, Participant 1, 43 years)

**Sub-theme 3.1: Support from management and the District Office:** The participants’ responses on support received from management and the district were mixed; some acknowledged getting support while others mentioned that although they got support, they felt that the support was partial and not enough. These mixed responses from different clinics revealed that support to clinics particularly from management was not uniform in the ICP:

‘When it comes to support from management and the mother hospital there is some support that we get, but there has been not much support from the district office.’ (PHC E, Participant1, 39 years)

‘The only support we receive is from other clinics but not district office.’ (PHC C, Participant 1, 28 years)

‘The support from district office has been good since implementation of ideal clinic.’ (PHC B, Participant 1, 38 years)

Organisational support is important in health care institutions and can make a considerable impact on boosting employees’ performance. Authors like Mughal (2019) attest to the fact that, perceived organisational support denotes the level and measure to which employees believe that their organisation recognises, supports and facilitates employees and their efforts. Therefore, supervisors, managers, and top management can foster employee performance by giving them support (Mughal 2019).

**Sub-theme 3.2: Support from each other and other clinics:** It is a common practice that staff in the healthcare institutions support each other. Occasionally, experienced and senior staff members support junior and inexperienced peers. The same applies to institutions that are underperforming where these receive support and benchmarks from those that are performing well. Some participants in the current study acknowledged this stating that the only support they got was from each other as staff members in the facility and/or from other neighbouring clinics. Their responses were as follows:

‘The only support we receive is from other clinics but not district office if it was not for that we will be nowhere with the ICP.’ (PHC F, Participant 1, 36 years)

‘At least we have a few clinics that are doing well and they allow us to benchmark from them.’ (PHC H, Participant 1, 26 years)

‘The problem is we are all in competition with each other and every clinic manager wants her clinic to be seen as the best otherwise, some staff members understand ICP very well and shame some of them do try to assist and mentor us.’ (PHC A, Participant 1, 40 years)

Peer relationships are another type of mentoring between individuals that serve the same functions as traditional senior–junior mentoring relationships and beyond. Several authors including Murrell, Blake-Beard and Porter (2021) recommend peer mentoring and support as the ideal strategy to improve performance. An important distinction between peer mentoring and support and traditional senior–junior mentoring relationships is reciprocity whereby traditional mentoring relationships are frequently conceptualised and measured as unidirectional while peer mentorships are described as either uniquely mutual or reciprocal which underscores the unique value provided by reciprocity within peer mentoring. Peer mentoring and support are indeed more readily available to individuals and may achieve a greater degree of communication, support, and collaboration than traditional hierarchical mentoring relationships while still providing the same range of career and psychosocial support functions as traditional mentoring relationships, including information sharing, career advice, exposure, coaching, and sponsorship, as well as emotional support, feedback, and friendship (Murrell et al. 2021).

## Discussion

The aim of the study was to explore and describe the experiences of nurses regarding the implementation of an ICP in eThekwini District, in KZN. The study identified that challenges related to the structure, process, and outcome were experienced by the nurses during the implementation of the ICP in eThekwini District. These findings concur with those of Muthelo et al. (2021) in their study on implementing the ICP at selected PHC facilities in Vhembe District, in Limpopo province where the challenges towards the successful and effective implementation of the ICP included poor implementation of the ICP, lack of resources, essential drugs, and equipment maintenance. The challenges experienced by the participants in the current study emerged during data analysis as the themes and sub-themes presented in the results section. The three themes included structural limitations, processes involved in the running of the clinic, and support offered to the PHC clinics.

### Structural limitations

Adequate infrastructure is needed for any existing health care system to provide service delivery timeously (Oyekale 2017). The findings from the current study revealed that structural limitations that existed in the PHC clinics posed major problems in ICP implementation. Tshililo et al. (2019) stated that since the integration of services in the PHC clinics there has been a great initiative in promoting holistic care. While on the one hand, the government is trying to integrate all services at the PHC level, the infrastructure remains a problematic issue in this regard. Kalonji and Mohamed (2019) attest that infrastructure can present challenges regarding the integration of some programmes in some PHC clinics. Poor maintenance of old infrastructure and the inability to provide sufficient funds in order to replace old structures are some of the factors that contribute to poor infrastructure (Oyekale 2017).

### Processes involved in the running of the clinic

A number of processes involved in the running of the clinic were not in place or not fully functional thus making it difficult to implement ICP successfully. Processes like guidelines and policies are supposed to be in place according to the ICP standards, but in many PHC clinics, they did not have these in place. This then made it difficult for them to keep up with the ICP standards.

Participants experienced a huge workload since the implementation of the ICP because there are a lot of changes that need to be made while at the same time experiencing staff shortages. According to Fagerström, Kinnunen and Saarela (2018), the increased workload in the health care sector consists of nurses performing duties which can be directly or indirectly linked to patient care. These duties vary from institution to institution according to many factors such as the physical layout of the facility or work processes. Dimunová et al. (2020) stated that nurses’ workload is a global phenomenon and it impacts on both the health of nurses and that of the patients. The environment that nurses work in predisposes them to various mental, physical, and behavioural risk factors and stress which is linked to the workload that they deal with from time to time. The stress that these nurses experience from increased workload further reduces the quality of their professional performance, resulting in stagnation in personal development and absence from the work environment.

Too much paperwork was also mentioned as a big concern, as well as information gathering being repetitive which wastes a lot of time during consultations. According to Anon (2018), although the majority of nurses acknowledge the importance of paperwork, others argue that paperwork requires them to put countless additional hours and to work through their breaks in order to get everything done. Furthermore, some administrative aspects that are expected from nurses on a daily basis mean that nurses have to stop actual patient care and focus on paperwork (Anon 2018). Paperwork is not only time consuming, but also does little to improve patient care. Often basic administrative duties are taken over by highly skilled nurses, who are then taken away from their nursing duties (Macphee & Dahinten 2017). In the long run, this is one of the elements that pushes nurses away from the profession.

Innovations in modern technology such as tablets, smartphones, among others, that nurses can use on the go to refer and report on patients can help in the reduction of paperwork. The health system can also improve the issue of too much paperwork by having guidelines in place that will help identify non-essential documents. The removal of non-clinical nursing documentation from the nursing remit can also be of great assistance in dealing with the problem. Unifying all patient documents into one patient document that can be functional for all health care providers is another strategy for tackling the problem. Finally, the implementation of an electronic system to reduce paperwork can be of great help (Okoye 2013).

### Support offered to the primary health care clinics

Mixed responses were gathered from the interviews concerning the support that participants perceive that they receive, tilting the scales unevenly among the participating clinics. Some participants mentioned that their main support is peer support received from either other PHC clinics or from each other as colleagues rather than managerial support from upper management and the district office. Others said that they do get support from upper management and the district office but even when they do, this support is not enough. Oyekale conducted an assessment study on PHC clinics’ service readiness in Nigeria post the year 2015 and discovered that there was a re-emphasis on equity and efficiency in health care delivery which was achieved through technical and financial support to health care clinics. The support that was offered was directly related to the realisation of several health-related targets in line with the sustainable development goals. The world’s major policy players like the World Health Organization (WHO) and World Bank have given support with regard to the resources to assist in some of these set goals (Oyekale 2017). Nevertheless, at times significant economic developments and the state of health care clinics in some countries are not aligned with global health development agendas (Oyekale 2017).

## Recommendations

The participants commented on several structural limitations that prevailed pertaining to staffing of the PHC facilities, infrastructure not being fit for purpose and a number of equipment issues. These limitations could be overcome by providing clinical practice guidance to nurses by making available systematically developed statements or processes to assist them with the type of clinical practice guidance determined by evidence-based criteria and clinical requirements which includes clinical policies, procedures, protocols, and guidelines (Ireland, National Clinical Effectiveness Committee 2015). The researchers recommend that policies and practice guidelines regarding sourcing and control of human and material resources be available and adhered to, in order to ensure standardised practice in resource provision. This could mitigate the prevailing challenges of infrastructure limitations.

Standard operating procedures (SOPs) provide details of the work processes that are to be conducted or followed and ensure adherence to best practices, consistency, proper orientation, and training to all personnel in an organisation (Padayachee & Munro 2020). Thus, it is recommended that SOPs be made available for all processes so that processes and procedures are standardised. The SOPs should include timelines and responsible persons in order to facilitate compliance by all parties. In-service education and orientation on any new developments in practice for example when new tools are introduced should be made available to all staff members to keep them abreast with new developments.

Support at work is an important component when it comes to enhancing employee performance. Employees who perceive greater organisational support tend to perform better (Mughal 2019). The nature of support and responsible persons to offer such support to staff should be included in policies and practice guidelines. This will ensure that support offered to staff is standardised and consistent (Padayachee & Munro 2020).

More studies on the implementation of ICPs should be undertaken on a wider scale either involving the entire KZN province or more provinces so that the findings and recommendations can be generalised to the whole country. Positive deviance studies are also recommended to facilitate learning from the PHC clinics that have successfully implemented the ICP.

### Strengths and limitations

The strength of the study lies in the fact that data were collected from the nurses who are the prime providers of PHC services and the implementers of an ICP thus allowing a provider-centric view of the implementation of ICP in eThekwini district. The limitations are that the study was conducted in one district and therefore the findings cannot be generalised to the whole of KZN or South Africa.

## Conclusion

The findings of the study confirmed that the nurses who are the drivers of the ICP have negative experiences regarding the implementation of ICP in eThekwini district. Several challenges prevail where there is little support offered to nurses when it comes to ICP implementation which could be responsible for some of the challenges experienced. Nurses’ negative experiences and perceptions make it difficult for them to accomplish the ICP standards. The researchers propose a number of recommendations which if instituted, could facilitate improved implementation of ICP and achievement of the 50% target in selected vital areas.
